# Coping strategies of patients with advanced lung or colorectal cancer in six European countries: Insights from the ACTION Study

**DOI:** 10.1002/pon.5259

**Published:** 2019-11-20

**Authors:** Lea J. Jabbarian, Ida J. Korfage, Branka Červ, Johannes J.M. van Delden, Luc Deliens, Guido Miccinesi, Sheila Payne, Anna Thit Johnsen, Mariëtte N. Verkissen, Andrew Wilcock, Agnes van der Heide, Judith A.C. Rietjens, on behalf of the ACTION consortium

**Affiliations:** ^1^ Department of Public Health Erasmus MC University Medical Center Rotterdam Rotterdam The Netherlands; ^2^ University Clinic for Respiratory and Allergic Diseases Golnik Slovenia; ^3^ Julius Center for Health Sciences and Primary Care University Medical Center Utrecht Utrecht The Netherlands; ^4^ End‐of‐Life Care Research Group, Department of Family Medicine and Chronic Care Vrije Universiteit Brussel and Ghent University Belgium; ^5^ Department of Public Health and Primary Care Ghent University Ghent Belgium; ^6^ Cancer Prevention and Research Institute Florence Italy; ^7^ International Observatory on End of Life Care, Division of Health Research, Faculty of Health and Medicine Lancaster University Lancaster UK; ^8^ Department of Psychology University of Southern Denmark Odense Denmark; ^9^ Department of Palliative Medicine Bispebjerg and Frederiksberg University Hospitals Copenhagen Denmark; ^10^ Department of Oncology University of Nottingham and Nottingham University Hospitals NHS Trust Nottingham UK

**Keywords:** advanced cancer, cancer, coping, oncology, end of life, psychology, support, tailoring

## Abstract

**Objective:**

Even when medical treatments are limited, supporting patients’ coping strategies could improve their quality of life. Greater understanding of patients’ coping strategies, and influencing factors, can aid developing such support. We examined the prevalence of coping strategies and associated variables.

**Methods:**

We used sociodemographic and baseline data from the ACTION trial, including measures of Denial, Acceptance, and Problem‐focused coping (COPE; Brief COPE inventory), of patients with advanced cancer from six European countries. Clinicians provided clinical information. Linear mixed models with clustering at hospital level were used.

**Results:**

Data from 675 patients with stage III/IV lung (342, 51%) or stage IV colorectal (333, 49%) cancer were used; mean age 66 (10 SD) years. Overall, patients scored low on Denial and high on Acceptance and Problem‐focused coping. Older age was associated with higher scores on Denial than younger age (*β* = 0.05; CI[0.023; 0.074]), and patients from Italy (*β* = 1.57 CI[0.760; 2.388]) and Denmark (*β* = 1.82 CI[0.881; 2.750]) scored higher on Denial than patients in other countries.

**Conclusions:**

Patients with advanced cancer predominantly used Acceptance and Problem‐focused coping, and Denial to a lesser extent. Since the studied coping strategies of patients with advanced cancer vary between subpopulations, we recommend taking these factors into account when developing tailored interventions to support patients’ coping strategies.

## 1 BACKGROUND

Patients diagnosed with advanced, incurable cancer commonly experience physical and psychological symptom burden.^1^, ^2^ When the disease has progressed to a point where curative treatments are unavailable, patients can particularly benefit from interventions aimed at improving quality of life.^2^

One way of assisting patients in the last phase of their lives is by supporting adaptive coping strategies. Coping strategies are distinct, ever changing cognitive, emotional and behavioral efforts to manage a (health) threat.^3^ For instance, when using denial, patients reduce the impact of their advanced disease by thinking that it is not real.^4^ Acceptance includes actively dealing with the advanced disease without unnecessary attempts to change the circumstances.^5^ Problem‐focused coping extends this towards a behavioral approach, for example, through taking actions to improve their way of living with their advanced disease.^4^ The use of coping strategies can vary between patients, situations, and over time.^4^ Different coping strategies may be used simultaneously or alternately.^6^ Whether a certain coping strategy is adaptive or not depends on the individual patient and situational context.^4^, ^7^, ^8^

Evidently, the way patients cope with their advanced disease impacts physical and psychological outcomes, such as quality of life and depressive symptoms.^2^ Since coping strategies are modifiable,^4^ supporting and encouraging adaptive coping strategies can contribute to the well‐being of patients, also when their disease has reached an advanced, incurable stage.^9^ Therefore, coping support is incorporated into interventions for patients with advanced cancer.^10^, ^11^ Coping strategies can also be used to tailor interventions. For instance, a trial compared a generic pain management program for older adults to programs matched to patients’ initial tendency towards either problem‐focused or emotion‐focused coping. Patients with problem‐focused coping were provided with specific instructions for relaxation and problem‐solving activities, patients with emotion‐focused coping received specific information on seeking support and expressing hope. The tailored interventions were more successful in reducing pain and symptoms of anxiety.^12^

The relevance of assessing and responding to coping needs throughout the disease trajectory of patients has been confirmed and recognized by numerous professional organizations, such as the American Society of Clinical Oncology^13^ and the National Institute for Clinical Excellence in the United Kingdom.^14^ Research in this area has mainly focused on patients in earlier stages of cancer.^15^, ^16^, ^17^ It is unclear if the findings in these patients are generalizable to patients with advanced cancer who face specific challenges, such as preparatory grief^18^ and increased existential distress.^19^

Given the importance of the sociocultural context for the appraisal of a (health) threat, it is not surprising that coping strategies have been found to differ among people with different socio‐cultural characteristics. For instance, younger people tend to use more active, problem‐focused coping than older people,^20^ and Korean Americans and Filipino Americans seemed to use more religious coping and escape avoidance than Caucasian Americans.^21^, ^22^ It is however unknown to what extent sociodemographic and clinical variables relate to the coping strategies of patients with advanced cancer. Detailed insights into coping strategies of patients with advanced cancer can inform both, the design of interventions delivering coping support and the evaluation and improvement of existing interventions by tailoring them to patients’ individual coping strategies.

We aimed to (1) characterize the prevalence of denial, acceptance, and problem‐focused coping among patients with advanced lung or colorectal cancer and (2) identify sociodemographic and clinical characteristics associated with the use of these coping strategies, including a comparison between countries.

## 2 METHODS

We used the sociodemographic and baseline data of patients of the ACTION trial, a cluster randomized trial investigating the effects of an advance care planning intervention compared to care as usual. We included only patients from the care‐as‐usual arm of this cluster‐randomized trial to avoid potential selection bias. Patients were recruited in outpatient pulmonology and oncology departments in academic and nonacademic hospitals in Belgium, Denmark, Italy, the Netherlands, Slovenia, and the United Kingdom, between June 2015 and May 2017 (see Box 1 for the inclusion and exclusion criteria).^23^ Written informed consent was obtained. Research ethics committees of the participating countries approved the trial.

**Box 1. Inclusion and Exclusion Criteria for the ACTION trial.**

**Table:** 

Inclusion criteria Histologically confirmed diagnosis of: Lung cancer: Small cell – extensive disease/ Stage III or IV* Non‐small cell – stage III or IV* Colorectal cancer: Stage IV or metachronous metastases*, *according to the 7^th^ edition of TNM classification and staging system Written informed consent to participate, WHO performance status of 0‐3 Exclusion criteria: Age <18 years, Unable to provide consent, Unable to complete questionnaire in country's language, Less than 3 months anticipated life expectancy, Taking part in a research study that is evaluating palliative care services or communication strategies.

### 2.1 Measures

#### 2.1.1 Sociodemographic and clinical variables

Patients provided information about their age, educational level, gender, living situation, and religion. Their healthcare providers registered type and stage of the disease and the time since diagnosis of the primary tumor and current stage of the disease, and on current treatment and performance status according to the World Health Organization (WHO) scale (0‐fully active to 3‐capable of only limited self‐care).^24^

#### 2.1.2 Coping

We measured patients’ coping strategies with the subscales Denial and Acceptance of the COPE Inventory and the subscales Planning and Active coping of the Brief COPE.^7^, ^25^ Patients were asked to rate the items according to the best description of how they had been coping with their disease during the past two months. Items were rated on a four‐point Likert scale from 1 (“I don't do this at all”), 2 (“I do this a little bit”), 3 (“I do this a medium amount”), to 4 (“I do this a lot”).

Following questionnaire instructions, we confirmed the subscales of the underlying coping strategies,^7^ by conducting a principal components analysis with the 12 selected items of the questionnaires. The analysis identified three distinct factors, each with eigenvalues above 1. The analysis confirmed the two subscales Denial and Acceptance.^7^ The subscales Active coping and Planning of the Brief COPE loaded on the same factor, in accordance with the structure of the questionnaire as described by the developers.^25^ We therefore combined Active coping and Planning, and, following previous research,^26^ labeled the resulting subscale “Problem‐focused coping” (see Box 2 for an overview of the subscales and included questions). For detailed psychometric information see Table S1. We summed the responses per subscale to create subscale sum scores. This resulted in a range of 4 to 16 for each subscale. Higher scores indicate more use of that particular coping strategy.

**Table 1:** Sociodemographic and clinical characteristics

	Belgium (n = 135)	Denmark (n = 68)	Italy (n = 139)	Netherlands (n = 168)	Slovenia (n = 25)	United Kingdom (n = 140)	Total (N = 675)
Age (years), mean (SD)	65.3 (9.5)	65.5 (9.0)	65.5 (9.6)	65.4 (8.1)	71.1 (9.5)	68.4 (11.0)	66.2 (9.6)
Years of education, mean (SD)	13.9 (4.4)	13.5 (5.9)	11.4 (5.2)	13.2 (3.7)	9.9 (3.3)	13.5 (4.7)	12.9 (4.7)
Gender (male), n (%)	91 (67.4)	35 (51.5)	90 (64.7)	111 (66.1)	10 (40.0)	70 (50.4)	407 (60.4)
Living with a spouse, n (%)	106 (79.1)	55 (80.9)	99 (73.9)	129 (78.2)	15 (62.5)	93 (69.9)	497 (75.5)
Having children, n (%)	114 (85.1)	62 (91.2)	118 (86.8)	146 (86.9)	21 (84.0)	60 (44.1)	583 (87.3)
Religion, n (%)							
Not specified	31 (23.8)	9 (13.6)	16 (11.7)	17 (10.1)	2 (8.0)	18 (13.2)	93 (14.0)
Not religious	30 (23.1)	38 (57.6)	24 (17.5)	76 (45.2)	2 (8.0)	58 (42.6)	228 (34.4)
Religious	69 (53.1)	19 (28.8)	97 (70.8)	75 (44.6)	21 (84.0)	60 (44.1)	341 (51.5)
Diagnosis, n (%)							
Lung cancer stage III/IV	79 (58.5)	34 (50.0)	71 (51.1)	76 (45.2)	0 (0.0)	82 (58.6)	342 (50.7)
Colorectal cancer stage IV	56 (41.5)	34 (50.0)	68 (48.9)	92 (54.8)	25 (100)	58 (41.4)	333 (49.3)
Years since diagnosis, mean (SD)	1.5 (1.7)	2.7 (3.2)	2.0 (3.5)	1.9 (1.9)	2.3 (2.4)	0.9 (1.4)	1.7 (2.4)
Years since diagnosis current stage, mean (SD)	1.1 (1.4)	1.6 (2.2)	0.8 (1.1)	1.2 (1.4)	1.3 (1.9)	0.4 (0.7)	1.0 (1.4)
Current systemic treatment,† n (%)	126 (96.2)	68 (100.0)	135 (97.1)	144 (86.2)	8 (53.3)	115 (87.8)	596 (91.6)
WHO performance status, n (%)							
3	0 (0.0)	0 (0.0)	0 (0.0)	2 (1.2)	1 (4.0)	5 (3.6)	8 (1.2)
2	7 (5.5)	1 (1.5)	2 (1.4)	12 (7.1)	13 (52.0)	20 (14.3)	55 (8.3)
1	56 (44.1)	40 (58.8)	65 (47.1)	122 (72.6)	10 (40.0)	49 (35.0)	342 (51.4)
0	64 (50.4)	27 (39.7)	71 (51.4)	32 (19.0)	1 (4.0)	66 (47.1)	261 (39.2)

SD = standard deviation

† Includes chemotherapy, immunotherapy, targeted therapy; treatments were not mutually exclusive.

Missings total: Age (n = 6), Education (n = 89), Gender (n = 1), Living with a spouse (n = 15), Having children (n = 6), Religion (n = 13), Years since diagnosis (n = 1), Years since diagnosis current stage (n = 6), Systemic treatment (n = 24), WHO performance status (n = 9).

**Box 2. Overview of the Subscales and items of the COPE and the brief COPE after the Principal Component Analysis.**

**Table:** 

Denial I act as though this hasn't even happened. I say to myself “this isn't real” I pretend that this hasn't really happened to me. I refuse to believe that this happened to me. Acceptance: I accept the reality of the fact that this has happened to me. I learn to live with my situation. I get used to the idea that this has happened to me. I accept that this has happened to me and that it can't be changed. Problem‐focused coping I concentrate my efforts on doing something about my situation. I take action to try to make my situation better. I try to come up with a strategy about what to do in my situation. I think hard about what steps to take in my situation.

### 2.2 Statistical methods

Missing items are common in palliative care trials.^27^ Given the low percentage of missing items (<5%) in our study, we carried out a complete case analysis by including only data of patients with full responses on all items of the three respective coping subscales.

We used SPSS 24 for the analyses. We summarized patients’ sociodemographic and clinical characteristics with means and standard deviations for continuous variables and counts and percentages for categorical variables. The distribution of scores on the coping subscales is presented with mean sum scores and standard deviations. Pearson correlation coefficients describe the linear correlation between coping strategies.

Multivariate multilevel regression models were used to analyze associations between coping strategies and sociodemographic and clinical variables. This type of model allows accounting for clustering at the hospital level and thus for nonindependency of observations.^28^ For inclusion numbers per hospital, see Table S2. The variable Country was included as a confounder. First, bivariate multilevel models were used to test associations between each sociodemographic and clinical variable and the distinct coping strategies. A significance level of *p* < 0.20 was used to select variables for the final model.^29^ For the final multivariate model, the significance level was set at *p* < 0.05. The Benjamini‐Hochberg procedure was used to account for the false discovery rate.^30^ Betas, 95% confidence intervals and Benjamini‐Hochberg *p*‐values are reported.

**Table 2:** Patient mean sum scores (SD) on coping subscales by sociodemographic and clinical characteristics

	Denial† (n = 655)	Acceptance† (n = 659)	Problem‐focused† (n = 643)
Mean Sum Score (SD)	6.6 (3.1)	12.6 (2.7)	12.2 (2.9)
Age in years			
18‐64	6.1 (2.8)	12.6 (2.9)	12.6 (2.7)
65‐79	6.9 (3.2)	12.6 (2.5)	12.0 (2.8)
≥80	7.3 (3.9)	13.3 (3.0)	11.5 (3.6)
Years of education			
0‐4	6.4 (2.8)	12.5 (2.2)	10.4 (3.3)
5‐12	7.0 (3.3)	12.3 (2.8)	12.2 (2.8)
≥13	6.1 (2.8)	12.9 (2.5)	12.3 (2.9)
Gender			
Male	6.6 (3.1)	12.5 (2.7)	12.0 (3.0)
Female	6.6 (3.2)	12.8 (2.8)	12.6 (2.7)
Living with a spouse			
Yes	6.6 (3.0)	12.6 (2,7)	12.2 (2.8)
No	6.7 (3.5)	12.6 (2.6)	12.1 (3.1)
Having children			
Yes	6.7 (3.2)	12.6 (2.7)	12.2 (2.9)
No	5.8 (2.5)	12.8 (2.7)	12.4 (2.9)
Religion			
Not specified	6.5 (3.0)	12.0 (2.9)	11.7 (3.0)
Not religious	6.3 (3.1)	12.7 (2.7)	12.2 (2.9)
Religious	6.9 (3.1)	12.7 (2.6)	12.5 (2.8)
Diagnosis			
Lung cancer stage III/IV	6.7 (3.1)	12.4 (2.6)	12.1 (2.8)
Colorectal cancer stage IV	6.6 (3.1)	12.8 (2.8)	12.3 (3.0)
Years since diagnosis			
≤1 year	6.5 (3.0)	12.7 (2.7)	12.3 (2.8)
> 1 year	6.5 (3.1)	12.8 (2.8)	12.4 (3.1)
Years since diagnosis current stage			
≤0.5 year	6.5 (3.0)	12.6 (2.6)	12.4 (2.6)
>0.5 year	6.7 (3.3)	12.7 (2.7)	12.0 (3.1)
Current systemic treatment‡	6.6 (3.1)	12.6 (2.6)	12.2 (2.8)
WHO performance status			
3	5.8 (2.4)	13.4 (2.1)	11.3 (1.9)
2	7.2 (3.6)	12.5 (2.8)	11.8 (2.9)
1	6.6 (3.1)	12.3 (2.7)	12.2 (2.8)
0	6.6 (3.0)	12.9 (2.7)	12.4 (3.1)
Country of residence			
Belgium	6.5 (2.9)	11.7 (2.8)	10.4 (3.0)
Denmark	7.6 (3.5)	13.3 (2.4)	12.6 (2.9)
Italy	7.5 (3.1)	12.5 (2.5)	12.8 (2.3)
Netherlands	6.0 (2.9)	12.5 (2.6)	13.0 (2.4)
Slovenia	7.3 (3.5)	12.6 (2.5)	12.4 (2.6)
United Kingdom	6.1 (3.0)	13.4 (2.8)	12.2 (3.2)

† Range for coping strategies is 4‐16 (higher scores indicate greater use of coping strategy).

‡ Includes chemotherapy, immunotherapy, targeted therapy; treatments were not mutually exclusive.

Missings range: Age (5‐6), Education (n = 79‐89), Gender (n = 1), Living with a spouse (n = 14‐15), Children (n = 6), Religion (n = 12‐13), Time since diagnosis (n = 1), Time since diagnosis current stage (n = 6), Systemic treatment (n = 20‐23), WHO performance status (n = 8‐9).

## 3 RESULTS

The analyses included 675 patients enrolled in the control arm of the ACTION trial. Numbers of patients per country ranged from n = 25 (Slovenia) to n = 168 (the Netherlands, Table 1). Patients’ average age was 66 (SD 9.6) years, the majority of patients were male (60%). Most of the patients were living with a partner (76%) and had children (87%), half of them described themselves as being religious (52%). Half of the population was diagnosed with lung cancer stage III or IV (51%). On average, patients were diagnosed with their primary tumor 1.7 years prior to inclusion (2.4 SD). Most patients received systemic antitumor treatment (92%) at time of inclusion.

### 3.1 Prevalence of coping strategies

Patients scored low on Denial (mean sum score 6.6 (SD 3.1) and high on Acceptance and Problem‐focused coping (mean sum score 12.6 [SD 2.7] and 12.2 [SD 2.9], respectively; Table 2). Higher scores on Acceptance were correlated with higher scores on Problem‐focused coping (*r* = 0.36; *p* < 0.001), higher scores on Problem‐focused coping were correlated with higher scores on Denial (*r* = 0.11; *p* < 0.001). Denial and Acceptance were not correlated (*r* = 0.04; *p* = 0.267).

### 3.2 Multilevel models

#### 3.2.1 Denial and sociodemographic and clinical variables

For Denial (n = 655), bivariate multilevel models showed significant associations (*p* < 0.20) with age, years of education, having children, years since the diagnosis of the primary tumor, and country of residence (Table S3). These variables were included in the final multivariate model (Table 3), which showed that older age was associated with higher scores on Denial than younger age (*β* = 0.05; 95% CI[0.023;0.074], *p* = 0.010), and patients in Italy (*β* = 1.57; 95% CI[0.760; 2.388]; *p* = 0.010) and Denmark (*β* = 1.82; 95% CI[0.881; 2.750]; *p* = 0.010) scored higher than patients in other countries.

**Table 3:** Multivariate multilevel analyses of coping strategies

	Denial (n = 655)			Acceptance (n = 659)			Problem‐focused (n = 643)			
	*β*	95% CI	*p*	*β*	95% CI	*p*	*β*	95% CI	*p*
Explanatory Variables										
Age	.049	.023, .074	.010*				−.016	−.041, .010	.0.324	
Years of education	−.042	−.095, .012	.233	.053	.005, .100	.143	.038	−.012, .088	..233	
Gender	NA			NA					.296	
Male							−.324	−.808, .158		
Female							Ref			
Having children			.204	NA			NA			
Yes	.673	−.065, 1.410								
No	Ref									
Religion	NA					.351			.351	
Not specified				−.550	−1.230, .127		−.387	−1.100, .326		
Not religious				−.083	−.585, .418		−.400	−.926, .130		
Religious				Ref			Ref			
Diagnosis	NA					.517	NA			
Lung cancer stage III/IV				−.181	−.667, .304					
Colorectal cancer stage IV				Ref						
Years since diagnosis	.041	−.058, .140	.496	.017	−.090, .124	.772	−.014	−.107, .079	.772	
Years since diagnosis current stage	NA			.156	−.030, .343	.211	NA			
WHO performance status	NA					.204			.057	
3				.882	−1.258, 3.022		−1.542	−3.816, .732	.183	
2				−.542	−1.523, .438		−1.335	−2.330, −.340	.009*	
1				−.591	−1.093, −.088		−.748	−1.268, −.228	.005*	
0				Ref			Ref			
Country of residence			.010			.204			.204	
Netherlands	.081	−.661, .823	.831	−.842	−1.897, .213		1.281	−.558, 3.120		
Belgium	.689	−.108, 1.486	.090	−1.881	−2.990, −.772		−1.679	−3.677, .319		
Slovenia	1.072	−.360, 2.499	.141	−.795	−2.334, .744		.798	−1.657, 3.253		
Italy	1.574	.760, 2.388	<.001	−.662	−1.770, .447		.518	−1.480, 2.517		
Denmark	1.812	.881, 2.750	<.001	−.186	−1.561, 1.190		.530	−1.936, 3.000		
United Kingdom	Ref			Ref			Ref			

* *p < 0.05*

#### 3.2.2 Acceptance and sociodemographic and clinical variables

Bivariate multilevel models for Acceptance (n = 659) showed significant associations (*p* < 0.20) with years of education, being religious or not, primary diagnosis, years since diagnosis of the primary tumor and diagnosis of the current stage, WHO performance status, and country of residence (Table S3). These variables were included in the final multivariate model (Table 3), which showed no significant associations.

#### 3.2.3 Problem‐focused coping and sociodemographic and clinical variables

For Problem‐focused coping (n = 643), bivariate multilevel models showed significant associations (*p* < 0.20) with age, years of education, gender, being religious or not, years since diagnosis of the primary tumor, WHO performance status, and country of residence (Table S3). These variables were included in the final multivariate model (Table 3). The association between the WHO status and Problem‐focused coping was borderline significant (*p* = 0.057) with patients with a WHO status of 1 (*β*=−0.75; 95% CI[−1.268; −0.228]) or 2 (*β*=−1.33; 95% CI [−2.330; 0.340], ie, somewhat restricted in activities) scoring lower on Problem‐focused coping than patients with a WHO status of 0 (ie, fully active).

## 4 CONCLUSIONS

Patients with advanced lung or colorectal cancer predominantly used Acceptance and Problem‐focused coping. The coping strategies were associated with sociodemographic and clinical characteristics.

Our findings that patients scored low on the use of Denial and high on Acceptance and Problem‐focused coping aligns with observations in patients with early stage cancer,^31^ patients recently diagnosed with incurable cancer^2^ and cancer survivors.^32^ Our results show that higher scores on Acceptance were correlated with higher scores on Problem‐focused coping. Endler and colleagues observed that patients with acute health problems predominantly used one coping strategy, in an effort to soothe their symptoms.^6^ Contrarily, patients with chronic health problems relied on more than one coping strategy, possibly because they have to adjust their life styles to a new situation.^6^ A similar challenge might be faced by patients with advanced cancer. Seemingly contradictory coping strategies Denial and Problem‐focused coping were positively correlated, be it only weakly. Denial includes pretending that the disease is not real while Problem‐focused coping concerns taking action to make a situation better.^4^ Possibly, both mechanisms are used to distract oneself from the actual situation. Denial itself has been association with negative and positive outcomes. One study in the United States of America showed that patients with asthma who scored high on denial tended to disregard symptoms of breathing difficulty, resulting in a higher rate of hospitalizations.^33^ Yet, in a study with patients with lung cancer, high scores on denial were related to a better overall perception of health and less pain.^8^

We found that older age was associated with higher scores on Denial than younger age. It has been hypothesized that older patients use “threat minimization” more, which includes keeping feelings to oneself and avoiding emotional distress by trying to forget.^34^ Patients in Italy and Denmark scored higher on Denial than patients in other countries. A review about culture and end‐of‐life care suggested a general reluctance to talk about death, as well as a trend towards partial/no disclosure of patients’ diagnosis and prognosis in Italy and Norway (a Scandinavian country with supposedly cultural resemblance to Denmark).^35^ This tendency was related to respect for privacy and/or to a strong death taboo,^35^ which might facilitate Denial as a way of pretending that the disease is not real. We also found borderline significant results that patients with a worse WHO performance status scored lower on Problem‐focused coping than patients who were fully active. The behavioral efforts often required for Problem‐focused coping might become more challenging with declining physical abilities.

### 4.1 Clinical implications

As Walshe et al stressed,^11^ a major conceptual issue in current interventions is that they largely ignore coping strategies of patients with advanced cancer, which might worsen their psychological experience.^11^ The clinical use of our results are twofold: (1) through tailoring interventions and framing discussions according to patients’ coping strategies and (2) through offering adequate coping support where needed. For instance, patients with a better WHO performance status might benefit from interventions activating their skills and confidence to manage their disease, acknowledging their tendency towards problem‐focused coping. For older patients, we advise to consider their tendency towards denial and adapt, for instance, information provision through carefully considering the detail of their information.^36^

Addressing coping strategies and providing support, eg, as an element of a palliative care intervention for patients with advanced cancer, is associated with improved quality of life.^9^ Our findings might help to decide which coping strategies to include in such interventions. Interestingly, our study showed that patients tend to use acceptance and problem‐focused coping more than denial. Referring to the approach‐avoidance dimension,^37^ we observed a higher tendency towards approach‐oriented coping than avoidance‐oriented coping. Early palliative care has been found to increase patients’ use of approach‐oriented coping strategies, leading to higher quality of life and lower depressive symptoms.^9^ Generic interventions offering approach‐oriented coping support might thus not be beneficial for most patients, instead interventions should focus on encouraging and building on pre‐existing strategies. To identify these pre‐existing strategies, as well as individual needs of patients, assessing coping strategies might be a useful approach. The Mental Adjustment to Cancer (MAC) scales is specifically developed for and widely used in cancer and palliative care settings and has shown good measurement properties.^38^, ^39^

### 4.2 Strengths

This paper presents unique data of patients with an advanced stage of two common cancer types in six European countries. We were able to collect detailed sociodemographic and clinical information, which allowed a thorough analysis of coping strategies.

### 4.3 Study limitations and recommendations

To minimize questionnaire burden, we restricted the assessment to three coping strategies. Future research should include additional coping strategies, such as spirituality or seeking social support, which might give more information about cultural sensitivity and relevance of coping strategies. In this study, we focused on patients with advanced lung or colorectal cancer. Replication of these findings in patients with other oncologic diagnoses and advanced diseases is recommended. Since we observed patients using a combination of different coping strategies, we recommend to investigate which combinations of coping strategies are beneficial for patients, and how these combinations might develop over time and between countries. Given that coping strategies are context‐dependent, it would be informative to determine which coping strategies were used to deal with which specific challenge. One should note that some beta weights as well as the differences between the mean sum scores were small, and replication of our findings is therefore desirable.

### 4.4 Conclusion

We investigated the prevalence of coping strategies and associated sociodemographic and clinical characteristics in patients with advanced cancer in six European countries. We found that patients with advanced cancer predominantly use Acceptance and Problem‐focused coping and use different strategies simultaneously. Denial was used less often. Being aware of the variance in the use of coping strategies can help healthcare professionals to fine‐tune their care. Interventions should be tailored to patients’ coping strategies.

## CONFLICT OF INTEREST

None declared.

## Supporting information


**Table S1.** Psychometric information for the coping scales
**Table S2.** Inclusion numbers per hospital
Table S3. Bivariate multilevel analysis of the association between sociodemographic characteristics, clinical characteristics and country of residence, and coping strategies (online only)

## Data Availability

The data that support the findings of this study are available from the corresponding author upon reasonable request.
