# Supplementary material for: Coping strategies of patients with advanced lung or colorectal cancer in six European countries: Insights from the ACTION Study
Source: Psychooncology. 2019 Nov 20;29(2):347–55. doi: 10.1002/pon.5259 (PMC7028098; doi:10.1002/pon.5259)
Supplement: Supplementary file 1 — Table S1. Psychometric information for the coping scales Table S2. Inclusion numbers per hospital Table S3. Bivariate multilevel analysis of the association between sociodemographic characteristics, clinical characteristics and country of residence, and coping strategies (online only) [file PON-29-347-s001.docx]

# APPENDICES

**S-Table 1.** Psychometric Information for the Coping Scales

|  | Cronbach’s $\alpha$ | Explained variance (%) | Factor loading |
| --- | --- | --- | --- |
| Denial | 0.856 | 29.413 |  |
| (1) I act as though this hasn’t even happened. |  |  | 0.738 |
| (2) I say to myself “this isn’t real”. |  |  | 0.883 |
| (3) I pretend that this hasn’t really happened to me. |  |  | 0.899 |
| (4) I refuse to believe that this happened to me. |  |  | 0.828 |
| Acceptance | 0.772 | 13.903 |  |
| (1) I accept the reality of the fact that this has happened to me. |  |  | 0.816 |
| (2) I learn to live with the situation. |  |  | 0.691 |
| (3) I get used to the idea that this has happened to me. |  |  | 0.801 |
| (4) I accept that this has happened to me and that it can’t be changed. |  |  | 0.698 |
| Problem-focused coping | 0.825 | 22.749 |  |
| (1) I concentrate my efforts on doing something about my situation. |  |  | 0.824 |
| (2) I take action to try to make my situation better. |  |  | 0.832 |
| (3) I try to come up with a strategy about what to do in my situation. |  |  | 0.810 |
| (4) I think hard about what steps to take in my situation. |  |  | 0.718 |

**S-Table 2.** Inclusion numbers per Hospital

|  | Number of included hospitals | Number of included patients |
| --- | --- | --- |
| Belgium | 2 |  |
| BE01 |  | 80 |
| BE02 |  | 55 |
| Denmark | 1 |  |
| DK01 |  | 68 |
| Italy | 2 |  |
| IT01 |  | 71 |
| IT02 |  | 68 |
| Netherlands | 3 |  |
| NL01 |  | 82 |
| NL02 |  | 68 |
| NL03 |  | 18 |
| Slovenia | 1 |  |
| SL01 |  | 25 |
| United Kingdom | 2 |  |
| UK01 |  | 74 |
| UK02 |  | 66 |

## S-Table 3. Bivariate Multilevel Analysis of the Association between Sociodemographic Characteristics, Clinical Characteristics and Country of Residence, and Coping Strategies (online only)

|  | Denial  (n=655) | | | Acceptance  (n=659) | | | Problem-focused  (n=643) | | | |
| --- | --- | --- | --- | --- | --- | --- | --- | --- | --- | --- |
|  | $\beta$ | 95% CI | *p* | $\beta$ | 95% CI | *p* | $\beta$ | 95% CI | | *p* |
| Sociodemographic Characteristic |  | | |  |  | |  | | | |
| Age in years | .051 | .026, .076 | <.001* | -.003 | -.024, .019 | .807 | -.031 | -.053, -.008 | .007* | |
| Years of education | -.063 | -.117, -.010 | .021* | .061 | .015, .108 | .010* | .048 | -.001, .097 | .057* | |
| Gender |  |  | .845 |  |  | .434 |  | | .024* | |
| Male | .049 | -.440, .537 |  | -.166 | -.581, .250 |  | -.499 | -.934, -.065 |  | |
| Female | Ref |  |  | Ref |  |  | Ref |  |  | |
| Living with a spouse |  |  | .503 |  |  | .740 |  |  | .548 | |
| Yes | -.188 | -.738, .362 |  | .080 | -.395, .555 |  | .153 | -.346, .651 |  | |
| No | Ref |  |  | Ref |  |  | Ref |  |  | |
| Having children |  |  | .011* |  |  | .462 |  |  | .480 | |
| Yes | .919 | .207, 1.631 |  | -.226 | -.829, .377 |  | -.230 | -.868, .408 |  | |
| No | Ref |  |  | Ref |  |  | Ref |  |  | |
| Religion |  |  | .344 |  |  | .153* |  |  | .101* | |
| Prefers not to specify | -.265 | -.993, .462 |  | -.612 | -1.237, .014 |  | -.493 | -1.145, .159 |  | |
| Not religious | -.397 | -.942, .148 |  | -.203 | -.671, .266 |  | -.475 | -.963, .012 |  | |
| Religious | Ref |  |  | Ref |  |  | Ref |  |  | |
| Clinical Characteristic |  |  | |  |  |  |  | |  | |
| Diagnosis |  |  | .535 |  |  | .092* |  |  | .822 | |
| Lung cancer, stage III or IV | .153 | -.331, .637 |  | -.358 | -.774, .060 |  | -.050 | -.488, .388 |  | |
| Colorectal cancer, stage IV | Ref |  |  | Ref |  |  | Ref |  |  | |
| Years since diagnosis | .081 | -.017, .180 | -.106* | .081 | -.004, .1660 | .060* | .082 | -.004, .168 | .061* | |
| Years since diagnosis of current stage | .078 | -.095, .252 | .374 | .196 | .048, .344 | .009* | -.025 | -.181, .131 | .750 | |
| Current systemic treatment^†^ |  |  | .751 |  |  | .374 |  |  | .484 | |
| Yes | .143 | -.742, 1.028 |  | .357 | -.430, 1.143 |  | -.289 | -.521, 1.100 |  | |
| No | Ref |  |  | Ref |  |  | Ref |  |  | |
| WHO performance status^‡^ |  |  | .245 |  |  | .043* |  |  | .012* | |
| 3 | -.233 | -2.422, 1.956 |  | -.163 | -2.033, 1.708 |  | -1.659 | -3.724, .405 |  | |
| 2 | .985 | .015, 1.955 |  | -.655 | -1.500, 1.900 |  | -.924 | -1.797, -.052 |  | |
| 1 | .149 | -.376, .674 |  | -.630 | -1.077,-1.834 |  | -699 | -1.173, -.225 |  | |
| 0 | Ref |  |  | Ref |  |  | Ref |  |  | |
| Country of residence |  |  | <.001* |  |  | .098* |  |  | .044* | |
| Netherlands | -.117 | -.814, .580 |  | -.945 | -2.126, .236 |  | .878 | -.549, 2.304 |  | |
| Belgium | .428 | -.307, 1.163 |  | -1.830 | -3.101, -.559 |  | -1.732 | -3.274, -.188 |  | |
| Slovenia | 1.240 | -.068, 2.548 |  | -.821 | -2.432, .789 |  | .228 | -1.689, 2.145 |  | |
| Italy | 1.401 | .670, 2.133 |  | -.946 | -2.217, .324 |  | .625 | -.921, 2.171 |  | |
| Denmark | 1.487 | .592, 2.383 |  | -.152 | -1.708, 1.404 |  | .449 | -1.445, 2.342 |  | |
| United Kingdom | Ref |  |  | Ref |  |  | Ref |  |  | |

^*^ *p<0.20,* and thus included in the final model.

^†^ Includes chemotherapy, immunotherapy, and targeted therapy.

^‡^ 0-Fully active, able to carry on all pre-disease performance without restriction, 1-Restricted in physically strenuous activity but ambulatory and able to carry out work of a light or sedentary nature, e.g. light house work, office work, 2-Ambulatory and capable of all selfcare but unable to carry out any work activities. Up and about more than 50% of waking hours, 3-Capable of only limited selfcare, confined to bed or chair more than 50% of waking hours.
